# Chewing Gums as a Drug Delivery Approach for Oral Health

**DOI:** 10.1155/2022/9430988

**Published:** 2022-06-20

**Authors:** Morteza Banakar, Sedigheh Moayedi, Erfan Shamsoddin, Zahra Vahedi, Mohammad Hasan Banakar, Seyyed Mojtaba Mousavi, Dinesh Rokaya, Kamran Bagheri Lankarani

**Affiliations:** ^1^Dental Research Center, Dentistry Research Institute, Tehran University of Medical Sciences, Tehran, Iran; ^2^Health Policy Research Center, Institute of Health, Shiraz University of Medical Sciences, Shiraz, Iran; ^3^Department of Pediatric Dentistry, Faculty of Dentistry, Shahed University, Tehran, Iran; ^4^Department of Orthodontics, Mashhad University of Medical Sciences, School of Dentistry, Mashhad, Iran; ^5^Cochrane Iran Associate Centre, National Institute for Medical Research Development (NIMAD), Tehran, Iran; ^6^School of Dentistry, Islamic Azad University Tehran Medical Sciences, Tehran, Iran; ^7^School of Dentistry, Yasuj University of Medical Sciences, Yasuj, Iran; ^8^Department of Chemical Engineering, National Taiwan University of Science and Technology, Taipei, Taiwan; ^9^Department of Clinical Dentistry, Walailak University International College of Dentistry, Walailak University, Bangkok 10400, Thailand

## Abstract

**Background:**

Drug delivery approaches with the shortest therapeutic period and the lowest side effects have always been considered a sublime target in the medical sciences. Among many delivery methods, chewing gum could be perceived as a promising drug carrier that can carry several types of drugs for oral health. These drug carriers could represent optimal therapeutic time and lower side effects due to their sustained release capability and lower required thresholds for the drug compared with other delivery approaches. The convenient use in the oral cavity's local environment and the ability to locally carry multiple drugs are considered the main advantages of this delivery approach.

**Aim:**

This review aimed to explore chewing gum as a promising drug carrier that can carry several types of drugs for oral health.

**Materials and Methods:**

Articles were searched for on PubMed, ISI, SCOPUS, Google Patents, the Royal Society of Chemistry website, and electronic databases using MESH terms and the following keywords: (“Gum” OR “Chewing gum”) and (“Drug delivery OR Drug delivery systems”) in the English language. No time limit was applied, and all documents as of August 30th, 2020 were retrieved.

**Results:**

Gum-drug interactions, mechanisms of release, and formulations of the drugs might all play a role in this versatile delivery method. Accordingly, chewing gum-based carriers may be presented as a plausible candidate for drug delivery in oral diseases.

**Conclusion:**

Gum-driven drugs could be introduced as promising candidates for treating oral diseases due to their ability to deliver the proper local dosages of active ingredients, short contact time, biocompatibility, and biodegradable chemical structures.

## 1. Introduction

The field of drug delivery is one of the most fascinating ones in medicine. Numerous medication delivery methods have been developed, sharing several advantages. The delivery process could make many advantages for patients, from the low period of therapy to less side effects due to their low usage dose [1]. Typically, the delivery approaches the target drug-loaded carriers and the routes for drug transmission. In recent decades, many families of carriers and their routes in the human body have been appropriately delineated and their advantages and disadvantages have been illuminated [2–4]. The polymeric and nanomaterial-based carriers with appropriate biocompatibility [5] and drug loading efficiency have attracted much attention for *in vivo* delivery in various cancer types. Noticeably, their toxicity is an issue that has remained in debate for many years. This challenge using delivery methods with a safe route is certainly promising [5–7]. Among the routes of administration, the oral types are attractive, mostly because of their comfortable appliances. As a result, approaches based on chewable carriers, such as drug-loaded gums and tablets, show some promise for many oral, esophageal, and GI-related diseases [8, 9]. Considering its unique characteristics, oral-based chewy delivery is highlighted as a promising candidate. Rapid onset of action, facile administration, low side effects, and appropriate local impact on oral diseases are all major factors contributing to this salience [9–11]. In this study, we discussed the characteristics of oral conditions for the effect of medications and the application of chewing gums in drug delivery for oral health. Additionally, medically applicable chewing gum types are further scrutinized in more detail.

## 2. Materials and Methods

In this narrative review, we searched PubMed, SCOPUS, Google Patents, the Royal Society of Chemistry website, and electronic databases using MESH terms and the following keywords: (“Gum” or “Chewing gum”) and (“Drug delivery” OR “Drug delivery systems”) with a language filter (English). No time limit was applied and all documents by August 30^th^, 2020 were retrieved. We did not use other filters. All articles and patents that satisfied our selection criteria were retrieved. After omitting the duplicates, we identified 30390 papers. Three independent reviewers assessed the article titles and abstracts, applying eligibility criteria. Articles were omitted if they were deemed irrelevant based on our keyword research. We defined the following criteria for inclusion using the PICO model:

  Population: There is no identifiable reference population.
  Intervention: Chewing gums are a vehicle for drug delivery in clinical trials, animal studies, and in vitro investigations.
  Comparison: Placebo-controlled or intra-individual pre-post comparison.
  Outcome: Cavity fighters, antibiotics, antibacterial, antifungal, antiviral, antiplaque, and remineralization are some clinical effects.

**Figure 1:**
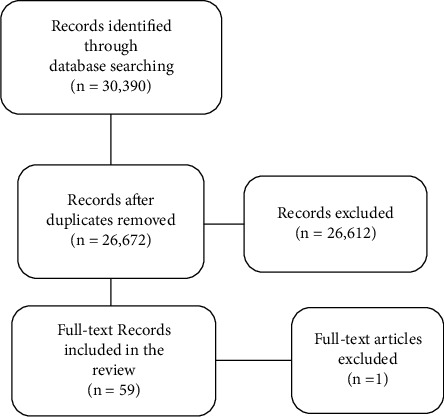
Study selection flow diagram.

References to these articles were also reviewed.  Figure 1 illustrates the procedure for conducting a literature search. After removing the duplicates, 26671 papers were obtained, of which only 60 studies made it through the eligibility assessments. One study was excluded after full-text reading and was deemed irrelevant to our inclusion criteria.

This study followed the recommendations of SANRA (a scale for the quality assessment of narrative review articles) to ensure internal consistency and proper presentation of the manuscript.

## 3. Results

### 3.1. Influencing Parameters in Chewing Gum-Based Drug Delivery

#### 3.1.1. Saliva Flow Rate

The accessibility of drug delivery is an essential factor in designing carriers. The oral cavity potentially provides systemic and local delivery accessibility. Saliva, a complicated multifunctional mixture that can solve the drug and delivery process's ability from the gum to the oral mucosa cells, acts as an intermediate platform. The saliva flow rate, which has been stimulated by chewing gum, has a positive impact on delivery; for instance, a study showed the beneficial effect of saliva flow on xerostomia [12]. Some reports have considered a plateau phase for saliva secretion rate while is being stimulated by chewing gum [11–13].

#### 3.1.2. Local Effect

Drug molecules released into the mucosal membranes during an equilibrium that occurred in minutes could be absorbed from the oral cavity microenvironment [14]. The buccal epithelium cells with a 20-40-cell thickness and a turnover of two weeks could play a vital role in the delivery process [15]. In the oral cavity, highly vascularized mucus membranes can provide an active drug circulation in jugular veins and act as a suitable drug reservoir [14]. The results showed that clearance is better in sublingual parts than in the labial vestibule. The main reason for these observations is the difference in the anatomy of the oral cavity [16–18]. The extended delivery time in the mouth causes the appropriate drug release rate and the maintenance of drug concentration for better therapy. Additionally, the local effects of therapeutic agents could be altered by the quality of drug distribution in the oral mucosa. The residence time of sublingual tablets showed the best time activity while chewing gum was better than the lozenge [19].

#### 3.1.3. Contact Time

Contact time is a significant determinant of the therapeutic period and side effects of the treatment regime. During chewing, the dissolution of ingredients occurs in the first few minutes of the process. Nevertheless, there is no standard time for chewing in general, but some case studies have suggested a 30-minute-period as a reasonable means of chewing time [20].

#### 3.1.4. Ingredients and Formulation

**Figure 2:**
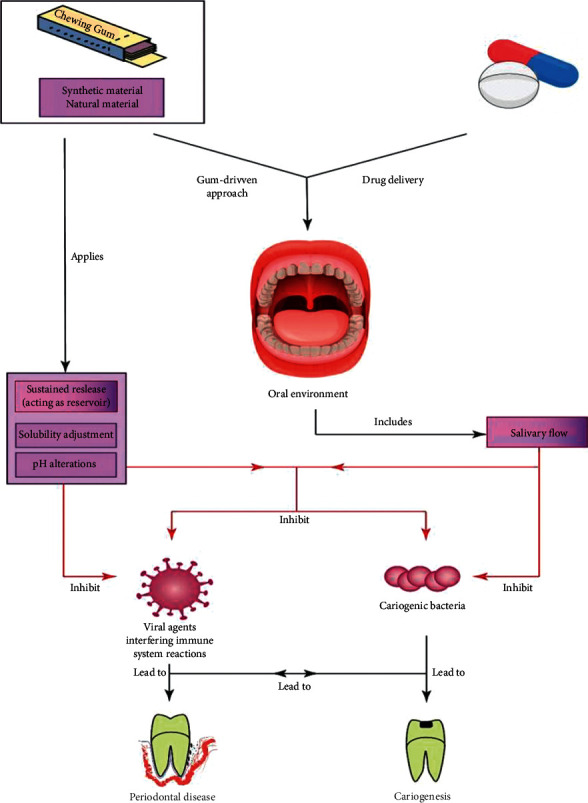
Schematic diagram of the gum drug delivery approach and factors affecting it.

When noticing drug formulation and active ingredients, hydrophobicity, stiffness, chemical structure, and interaction types are essential. The hydrophobicity/hydrophilicity of gum influences the drug-gum interactions and reflects the quality of interactions between the drug and gum structures [21]. The hydrophilicity of carriers in delivery could be mentioned as one of the most important physical properties that potentially control the drug release rate and mechanism. Furthermore, for chewing gums, this parameter could be impressive because the release process closely depends on the hydrophilicity state of the drug-containing carrier [22]. The cyclic oligosaccharides with dual hydrophobic and hydrophilic structures provide excellent opportunities for loading hydrophobic drugs. This could boost the drug's solubility in saliva by its hydrophilic entity. Agglomeration and encapsulation with biocompatible polymers could be other approaches to the formulation [21, 23]. The interaction between drugs and gum structures influences the release time and the mechanism of drug delivery. While water-soluble drugs need 10–15 minutes for a significant release, some lipid-soluble drugs may require more time. Finally, the pH of saliva is another important property that could greatly impact how drugs are released [21, 23].  Figure 2 shows the schematic diagram of the gum drug delivery approach and the factors affecting it.

### 3.2. Chewing Gum Material Base

Masticatory resins, which resemble chewing gum, date all the way back to the Bronze Age, some 5000 years. In Finland and Sweden, resin particles have even been found with teeth evidence [24]. Chicle from the Sapodilla tree was sold like chewing gum for the first time in 1848. In 1869, Ohio doctor William F. Semple patented chewing gum as a sweet and a medication to protect teeth. Aspergum^®^, the first medicated chewing gum introduced in the United States in 1924, was the first to gain acceptance as a drug delivery system through the market release of nicotine chewing gum [11, 25]. On the other hand, modern chewing gums are frequently made from synthetic resins, as opposed to the natural latex basis used by Thomas Adams, who obtained the first patent for a chewing machine to produce chicle kneaded and smoothness. In 1991, the European Communities Commission allowed the word “chewing gum” as a medicinal dosage form in guidelines [11, 26, 27].

With extensive usage in the pharmaceutical and food industries, polyol sweeteners are generally used as substitutes for sugar and alcohol. Because of their laborious metabolizing processes in the body, they could not act as a calorie source. They could be used in food and as a drug carrier when treating oral diseases. Xylitol and sorbitol have been broadly evaluated as noncariogenic gum-based materials and agents for preventing lactic acid production by bacteria in the mouth environment. Preventing lactic acid formation could decrease dental and oral diseases such as dental caries [28]. These protective results are partially attributed to the fact that chewing xylitol and sorbitol-based gums, even for five minutes per day, could significantly reduce plaques and *S. mutans* levels [29,30]. *Streptococcus mutans* could not metabolize xylitol, which could result in bacterial accumulation in intercellular spaces and competition for sucrose, a critical substrate in bacterial metabolic pathways [31–33].

Alginate-based gums are another candidate. The crosslinking process using calcium ions can control drug release profiles in these gums. Other than altering the release rate, this further could increase the interactions between the alginate and the drug [34]. Nowadays, natural and synthetic substances with significant therapeutic properties, such as xanthan, chitosan, and gellan, have been considered for chewing gum [35]. Some of these materials are listed in Table 1.

### 3.3. Types of Medicaments That Can Be Used in Chewing Gums

To date, various drugs and substances with various therapeutic properties have been introduced for use in chewing gum, some of which and their therapeutic properties are listed in Table 2. These materials based on the source are divided into synthetic and natural, which are mentioned below.

#### 3.3.1. Synthetic Material

Various types of synthetic gum-driven agents have been suggested so far. The ingredients that fight cavities include Ca phosphate [36–39] and bicarbonate [40, 41]. They also include chlorhexidine [42] and copper chlorophyllin [43]. Hydrogen peroxide [44] and zinc [45] are antibacterial and antiviral agents, as are pycnogenol [46], stannous EDTA [47], sulfathiazole [11], urea [48, 49], zirconium silicate [50–52], and also fluoride [53–56]. Among these drugs, chlorhexidine, hydrogen peroxide, sulfathiazole, and zinc have more potential to be loaded in chewing gum and as an oral disease remedy [11]. Chlorhexidine was used in nanocapsules to treat dentin substrates that had been decalcified. Gum could be used as a carrier for this drug [57].

#### 3.3.2. Natural Material

Some natural ingredients can be used in chewing gum with medical properties. Garlic, for instance, shows antiviral, antibacterial, and antifungal properties [58]. Ginger can also counteract respiratory viruses [59]. Oregano has powerful antiviral properties. In high concentrations, it could inactivate viral agents within one hour of exposure [60]. Lemon balm and green tea have antiviral properties and effectively against various viruses, including influenza, herpes, adenovirus, and HIV [61, 62]. Elderberry exhibits antiviral and antibacterial effects and is considered a remedy for the common cold in traditional medicine [63]. Coconut oil has also shown strong antiviral properties. It can either eradicate or inactivate harmful bacteria in the body [64]. Black walnut has antiviral, antifungal, antimalarial, and antiparasite properties [65]. Turmeric could inhibit viral replication and interfere with the virus-cell binding process [66].

### 3.4. Chewing Gums as Drug Carriers for Oral Health

**Figure 3:**
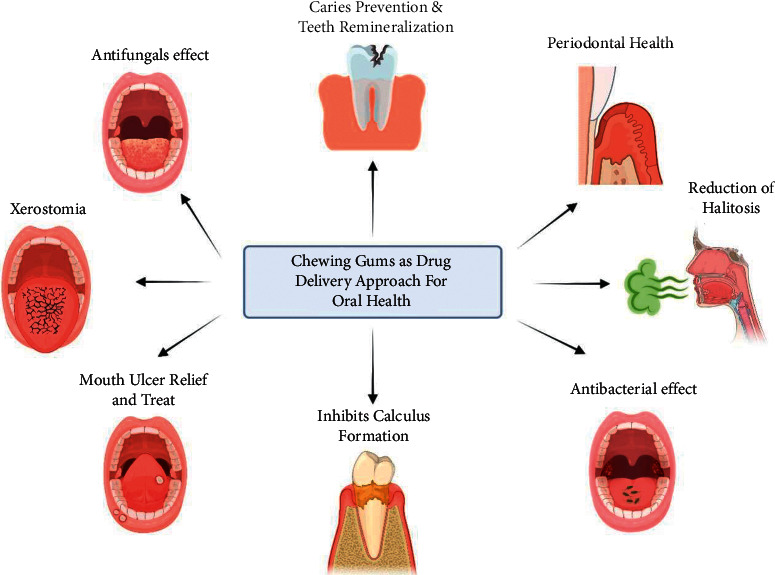
Chewing gums as a drug delivery approach for oral health.

Chewing gum can be used to provide a controlled dose of an active component to the mouth. Chewing gum active compounds are released in a variety of ways, depending on parameters such as chewing speed, gum base concentration, and active ingredient solubility in water, allowing them to remain in the mouth for a longer period of time. Chewing gums could transport chlorhexidine, calcium, and carbamide-based medications such as captopril, nitroglycerin, methadone, antihistamines, and antifungal-based compounds as drug carriers [67–69].  Figure 3 illustrates some of the uses of chewing gum as a drug delivery approach for oral health.

Mouth dryness, also known as xerostomia, is a condition that occurs when salivary glands do not produce enough saliva to keep the mouth moist. Chewing gum has long been recognized to increase saliva production. The first five minutes of chewing generate a 10-fold increase in salivary flow over unstimulated salivation [70]. Since gum chewing has been shown to alleviate the symptoms of xerostomia in certain conditions, such as Sjogren's syndrome, various clinical experiments have been conducted to support this claim. With pilocarpine added to the chewing gum, salivary secretion can be boosted to an even greater extent. Saliva has a buffering capacity and may be able to lower the acidity of the stomach juice. Gum without active ingredients has been shown to prevent postprandial reflux in clinical trials. In order to get the most out of this, an antacid should be added to the gum.

Gum chewing stimulates the flow of saliva. Mechanical mastication is considered a key element in this boosting effect. Findings show that chewing gum could potentially decrease gingivitis and carious lesions. Additionally, calcium-containing gums could remineralize incipient caries [71]. Tooth plaques are the most critical risk factor affecting dental and periodontal health. Periodontal disease could be partly treated by sodium bicarbonate/sorbitol mixture-based gums. These types of gum could increase the pH of saliva and reduce dental plaque accumulation [72]. Patients with weakened immune systems are more likely to develop bacterial or fungal infections of the mouth. Dental and oral infections can be alleviated by chewing chlorhexidine gum. Dental plaques can be treated with chlorhexidine and decapeptide-based antiseptic gums. Chlorhexidine/xylitol-based gums have the potential to significantly lower the load of S. mutans and lactobacilli in the mouth. [73–76]. Since the harsh taste of chlorhexidine in mouthwash is easily disguised by the sweet flavor of chewing gum, it is a better choice for daily oral hygiene than a chlorhexidine mouthwash [77].

Chewing gums with antibacterial actions in the oral cavity, such as gramicidin and neomycin, are similar to sulfathiazole chewing gums. For Vincent's illnesses, metronidazole gums could substitute penicillin-loaded gums in terms of bacterial resistance. The miconazole chewing gum has been demonstrated to be at least as effective as a miconazole oral gel in treating oral candidiasis in clinical trials involving patients. In addition, patients favored chewing gum over oral gel since it was more convenient and had fewer adverse effects [78–80]. Namibian chewing gum, which contains *Diospyros lycioides,* indicates an antibacterial effect on *Streptococcus mutans* and *Streptococcus sanguis* [81, 82]. Another natural ingredient used in gum is the magnolia bark extract, which shows an antibacterial effect against *Porphyromonas gingivalis*, *Fusobacterium nucleatum*, and *S. mutans* [83].

Bacterial colonization has long been claimed to be prevented by fluoride-containing chewing gums. For example, fluoride ions block the metabolism of plaque bacteria when used in a dental therapy, as might other chemicals with therapeutic applications that act locally or are absorbed through the oral and buccal capillaries. Patients with xerostomia and children in fluoride-deficient areas, as well as adults with a high incidence of dental caries, may benefit from chewing fluoride-containing gum. Adults can also benefit from its use in preventing dental cavities [53–56].

Other commercially available options include vitamin C chewing gums and tablets. Chewing vitamin C-fortified gum at least five times a day for three months reduced the production of supragingival calculus in comparison to a control gum and no chewing gum at all. Calcium phosphate deposits are thought to be reduced because of the acidic characteristics of vitamin C [84]. However, dental enamel damage due to high local vitamin C concentrations is the main drawback of these gums [85]. Chewing gum with pyro/triphosphate supplementation showed similar benefits after six weeks of use, which may be due to the calcium-sequestering activities of polyphosphates on the enamel. However, the reduction in calculus formation was only observed on supragingival surfaces [86].

Oral health is becoming more and more concerned with appearance, notably the appearance of white teeth. Stains caused by chromogens from food, drink, or smoking can be extrinsic or intrinsic, depending on the source (or calculus). Polyphosphates have been added to sugar-free chewing gums to help prevent and remove extrinsic stains. In short-term (2 days) trials, a sugar-free gum containing sodium hexametaphosphate outperformed a control gum at reducing stain formation [87, 88].

Oral malodor is caused by anaerobic gram-negative bacteria adhering to the tongue or associated with periodontitis, which produces VSCs such as hydrogen sulfide and methyl mercaptan [89]. Gums that contain active compounds that target bacteria that cause bad breath have been shown to reduce the amount of VSCs in the mouth and the amount of VSCs in the mouth. To reduce VSC levels after chewing, the zinc-allyl isothiocyanate combination works particularly well because of zinc's affinity for sulfur compounds [90, 91]. To reduce oral odor, chewing gum with the magnolia bark extract or eucalyptus essential oil has been successful when paired with zinc, which inhibits the viability of the bacteria that produce VSC [91, 92].

Probiotics (*L. reuteri, L. salivarius, and L. plantarum*) have been studied to minimize dysbiosis and maintain a balanced microbiota in the form of chewing gum. Because of the prevention of antibiotic adverse effects, probiotics are also indicated as a supportive treatment alongside scaling and root planning [93].

Minor pain treatment can benefit from the use of chewing gums as a drug delivery mechanism because of its rapid onset of action and reduced risk of digestive side effects. Up to 63% of the normal dose of acetylsalicylic acid can be delivered by chewing an acetylsalicylic gum for 15 minutes. Drug absorption rates are faster in the liquid form compared to a tablet form with the same dosage of the same medications. Toothaches could possibly be relieved faster if drugs had a faster absorption rate [94–96].

There are various limitations to using chewing gum as a medicine delivery device, including the fact that it prevents you from eating, drinking, and conversing while you have a delivery system in your mouth. Due to saliva dilution, the mouth cavity always shrinks, and the medicine secreted in saliva soon disappears as a result of the swallowing process. When it comes to the release of drugs from chewable formulations, chewing habits have been shown to have a substantial impact.

### 3.5. Future Trends

The science of using different carriers as a drug delivery system is advancing daily. Chewing gum, mousse [97], exosomes [98], and micro- and nanorobot [99] have been considered. More attempts will be made in the future to develop drugs that use chewing gum as a drug delivery mechanism. The treatment of fungal infections, prevention of cavities and other dental health problems, remineralization of teeth, cold relief, increased energy, antinausea, and a slew of other benefits of this novel drug delivery technique are all likely to play a key role in future research. Chewing gum does, in fact, take some time to gain acceptance as a method of drug delivery. Alternative delivery mechanisms for administering pharmaceuticals locally to the oral cavity may be replaced by medications incorporated into chewing gums. The reason is simple: the chewing gum administration system is convenient, easy to deliver anywhere, at any time, and its pleasant taste encourages patient compliance, particularly among children.

## 4. Conclusions

Gum-driven drugs could be introduced as promising candidates for treating oral diseases. This is due to their ability to deliver the proper local dosages of active ingredients, short contact time, biocompatibility, biodegradable chemical structures, and ability to maintain a state of eubiosis. These benefits have spurred many people to research to make a lot of different kinds of medicated chewing gum commercials.

## Figures and Tables

**Table 1:** Carriers that can be used in chewing gum and their properties.

Carriers	Origin/components	Properties
*Acacia*	Stems of tree *acacia arabica*	Antimicrobial activity
Alginic acid	Natural polysaccharides isolated from the brown seaweed	Antianaphylaxis effect, immunomodulatory activity, and antioxidant activity
Dextrin	Produced by the hydrolysis of starch and glycogen	Applications as a targetable carrier and bioadhesive
Gelatin	Linear anionic high molecular weight exopolysaccharide, commercially produced by microbial fermentation	Antibacterial drug delivery systems
Guar	Biopolymer extracted from the seeds of *Cyamopsis tetragonolobus* beans (*Leguminosae* family)	Sustained-release systems
Lecithin	The mixture of fats that are essential to cells, derived from sunflower seeds, eggs, or soybeans	Oral and aerosol delivery systems
Sodium alginate	Brown seaweeds (*Phaeophyceae*)	pH-sensitive carrier
Xanthan	Hydrophilic, anionic-bacterial heteropolysaccharide, derived from the fermentation of gram-negative bacteria *Xanthomonas campestris*	Antioxidant, anti-inflammatory, antibacterial, and biofilm inhibitor
Gellan	Exocellular polysaccharide secreted by *Pseudomonas elodea*	Anti-inflammatory
Rosin	Clear, pale yellow to dark amber thermoplastic resin present in oleoresins of the tree *Pinus roxburghii* and *Pinus taeda*	Film-forming, coating properties, and sustained and controlled drug release systems
Chitosan	Invertebrates, insects, and yeast	Antifungal, wound healing acceleration, and immune system stimulation
Tamarind seed polysaccharide	Galactoxyloglucan, tamarind seed polysaccharide	Noncarcinogenicity, mucoadhesive nature
Carrageenan	Extract from a red seaweed commonly known as Irish moss	Immunomodulatory and antioxidant activity
*Terminalia catappa*	*Terminalia catappa* leaves	Antimicrobial, antioxidant, anticancer, antiviral, anti-inflammatory properties
Pectin	Methoxyester of pectic acid derived from plant cell walls	Anticancer, immunostimulation, anti-inflammatory, antibacterial, antiadhesive effects

**Table 2:** Therapeutic effects and materials used in chewing gum on oral health.

Therapeutic effect	Material used
Analgesic	Aspirin, benzocaine, and eugenol
Acid neutralization	Antacid, calcium carbonate, carbamide, bicarbonate, xylitol
Antiplaque (biofilm control)	Chlorhexidine gluconate, eucalyptus, mastic, xylitol, sorbitol sulfonamides, neomycin, gramicidin, hydrogen peroxide, zinc, sulfathiazole, magnolia bark extract, fluoride, and propolis
Anticalculus formation	Vitamin C and polyphosphates
Antioxidant, antiseptic, and healing	Green tea and aloe vera
Dental caries prevention	Fluoride, calcium phosphate, bicarbonate, copper, chlorophyllin, and xylitol
Antibacterial agent	Chlorhexidine gluconate, sulfonamides, neomycin, gramicidin, hydrogen peroxide, zinc, sulfathiazole, fluoride, and propolis
Antiallergy	Cetirizine, diphenhydramine hydrochloride
Gingival inflammation	Green tea, amyloglucosidase combined with glucosidase, egg-white lysozyme, and rhozyme P-11
Deficiency of vitamin C	Vitamin C
Plaque removal	Zirconium silicate, decapeptide-based antiseptic, and sodium bicarbonate
Dental enamel strengthening agent	Potassium aluminum sulfate, calcium, CPP-ACP, and fluoride
Oral candidiasis	Miconazole, nystatin
Periodontal disease	Sodium bicarbonate/sorbitol
Vincent disease	Metronidazole
Reduction of planktonic bacteria in saliva	Chlorhexidine, xylitol, chitosan, mastic, magnolia bark extract, and propolis
Mitigation in a volatile sulfur compound	Eucalyptus, zinc, and magnolia bark extract
Tooth stain prevention	Polyphosphates and hydrogen peroxide

## Data Availability

The data used to support the findings of this study are available from the corresponding author upon request.
